# Neonatal Adrenal Haemorrhage Mimicking Acute Scrotum: A Case Report

**DOI:** 10.15190/d.2025.9

**Published:** 2025-06-30

**Authors:** Muhammad Mudasir Saleem, Mishal Pervaiz, Ismail Mazhar, Uswah Shoaib, Muhammad Umar Rafique

**Affiliations:** ^1^Paediatrics and General Surgery, Combined Military Hospital, Lahore, Pakistan; ^2^Department of Anaesthesiology, Punjab Rangers Teaching Hospital Lahore, Pakistan; ^3^CMH (Combined Military Hospital) Lahore Medical College and Institute of Dentistry, Lahore, Pakistan

**Keywords:** Neonatal adrenal haemorrhage, acute scrotum, case report, testicular torsion, ultrasonography, retroperitoneal bleeding.

## Abstract

Acute scrotum in neonates is a rare condition with multiple causes, including incarcerated hernia, testicular torsion, birth trauma, gross hydrocele, and neonatal adrenal haemorrhage, the least common aetiology. Early diagnosis and intervention are essential to prevent testicular ischemia. Due to the continuity between the retroperitoneum and the scrotum via the processus vaginalis and inguinal canal, blood from an adrenal haemorrhage may track down into the scrotum, leading to swelling and discoloration. We report a case of a 1-day-old male neonate born via emergency caesarean section at 37 weeks due to foetal distress. The baby initially admitted to the NICU for transient tachypnoea, developed a right hemi-scrotal swelling with bluish discoloration on the second day of life. Scrotal ultrasound suggested testicular torsion, but Doppler imaging showed absent blood flow. Further abdominal ultrasound confirmed a right adrenal haemorrhage. The neonate was managed conservatively with intravenous fluids, antibiotics, oxygen support, and coagulation management. Serial ultrasounds showed gradual resolution, and he was discharged on the 17th postnatal day. Follow-up at 1 and 3 months showed complete recovery with normal growth. Neonatal adrenal haemorrhage should be considered in cases of acute scrotum, especially in neonates with birth asphyxia. Abdominal ultrasound can aid in diagnosis, preventing diagnostic delays, unnecessary surgery, and anaesthesia exposure. This case highlights the importance of thorough imaging and awareness of rare differential diagnoses, contributing to improved clinical practice and better neonatal outcomes.

## INTRODUCTION

Neonatal adrenal haemorrhage (NAH) is a rare condition, with an incidence of approximately 0.2% to 0.5% ^1^. Risk factors include birth asphyxia, hypotension, sepsis, and perinatal distress ^2^. Term male neonates are more frequently affected due to their higher birth weight, and the right adrenal gland is particularly vulnerable because of its anatomical position between the liver and spine, making it more prone to compressive forces during delivery ^3^. The clinical presentation of NAH is variable, ranging from asymptomatic cases to life-threatening hypovolemic shock due to adrenal insufficiency ^4^.

A rare but clinically significant presentation of NAH is scrotal hematoma, which can mimic acute scrotum, leading to potential misdiagnosis as testicular torsion ^5^. Only a limited number of cases with this presentation have been reported in the literature. We present a rare case of a neonate with acute scrotum who underwent surgical exploration for suspected testicular torsion but was later diagnosed with NAH through retrospective abdominal ultrasonography following intraoperative findings of bilateral scrotal hematoma. This case highlights the importance of thorough preoperative evaluation, particularly in neonates with risk factors for NAH, to prevent unnecessary surgical intervention. This manuscript was prepared following the CARE guidelines (https://www.care-statement.org).

## CASE REPORT

**Figure 1:**
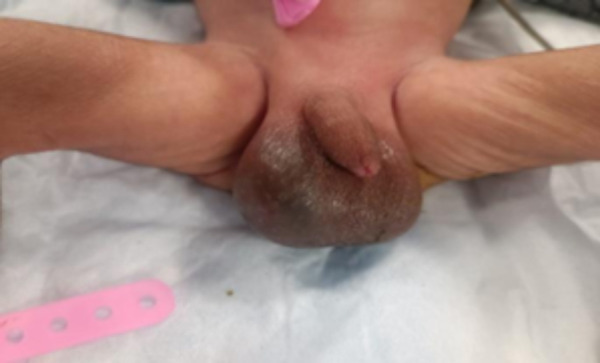
Initial presentation with bluish scrotal discoloration on right side

A paediatric surgery consultation was requested for a 1-day-old baby boy in the neonatal intensive care unit (NICU) at a tertiary care centre in Lahore. The baby was delivered via emergency caesarean section at 37 weeks of gestation due to foetal distress to a non-consanguineous couple. His mother was a primigravida who had initially undergone a trial of spontaneous vaginal delivery, which failed to progress. At birth, the baby weighed 2700 grams, with APGAR scores of 5, 7, and 8 at 1, 5, and 10 minutes, respectively. Family history was unremarkable, and the parents had no history of psychosocial disturbances. There were no known hereditary conditions or genetic disorders in the family. He required initial resuscitation with AMBU (artificial manual breathing unit) ventilation and was admitted to the NICU for tachypnoea. On the second day of life, the resident doctor noted right hemi scrotal swelling with discoloration and ordered an ultrasound. The ultrasound raised suspicion of testicular torsion, and Doppler ultrasonography confirmed the absence of ipsilateral testicular blood flow. At this point, paediatric surgery was consulted. On evaluation, the baby had a pulse of 136 beats per minute, blood pressure of 68/52 mmHg, a temperature of 98.3°F, and a respiratory rate of 70 breaths per minute. He was receiving oxygen support via nasal prongs for transient tachypnoea of the newborn. Abdominal examination was unremarkable. Groin examination revealed bilateral intact inguinal orifices, while the right testis was swollen, firm, and exhibited bluish discoloration of the scrotal skin (Figure 1).

**Figure 2:**
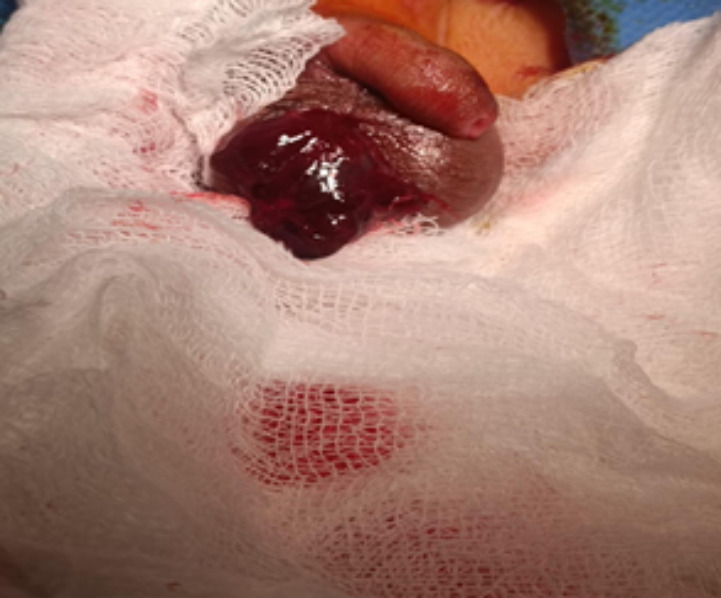
Gross right scrotal hematoma on exploration

The contralateral testis appeared normal. Based on history, clinical findings, and Doppler scan, a diagnosis of neonatal testicular torsion on the right side was made. Parents were counselled in detail and informed written consent was obtained. Exploration was done through midline approach. Right-sided gross testicular hematoma was found with normal underlying testis and epididymis (Figure 2).

**Figure 3:**
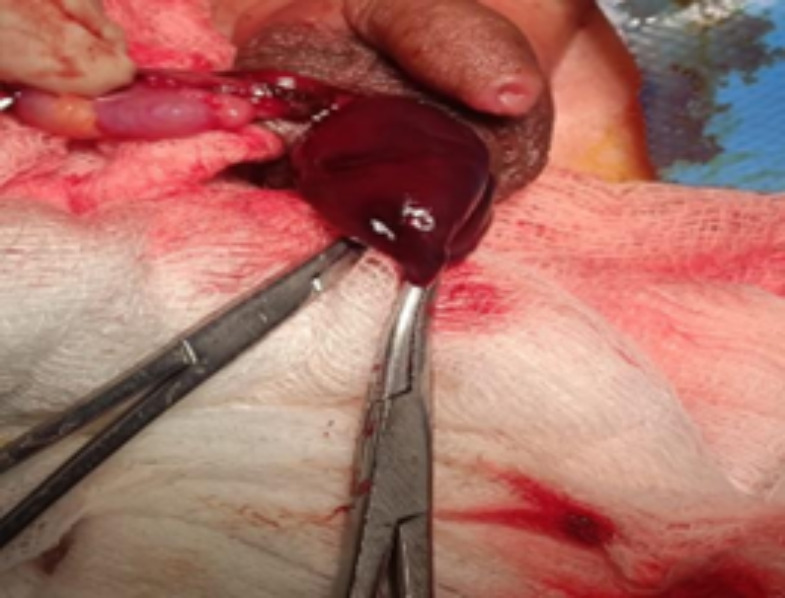
Left scrotal hematoma on exploration of the left hemi scrotum through midline wound with normal right testis

There was Perioperative suspicion of hematoma on the left side, for which left testicular exploration was also done. Left hemi scrotum also revealed scrotal hematoma, which was comparable to the right side with normal underlying testis (Figure 3).

**Figure 4:**
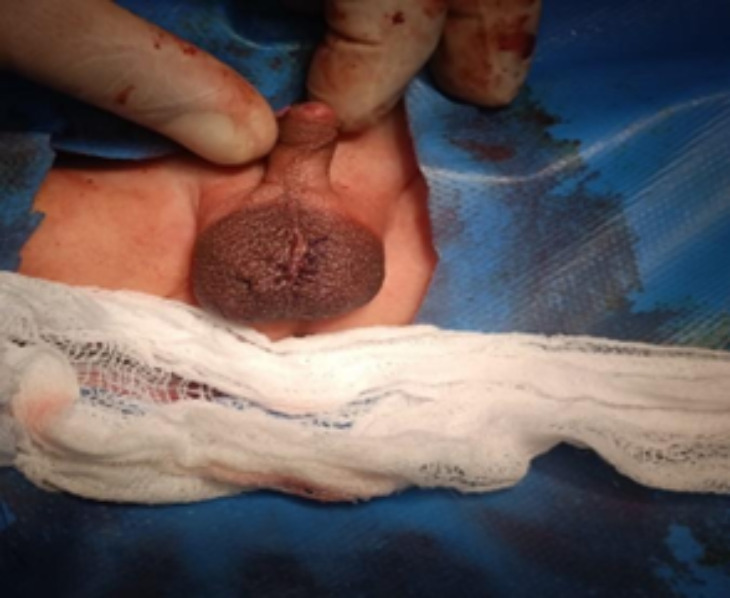
Final presentation on completion of surgery

Scrotal hematoma evacuation was performed, followed by wound closure in layers (Figure 4).

**Figure 5:**
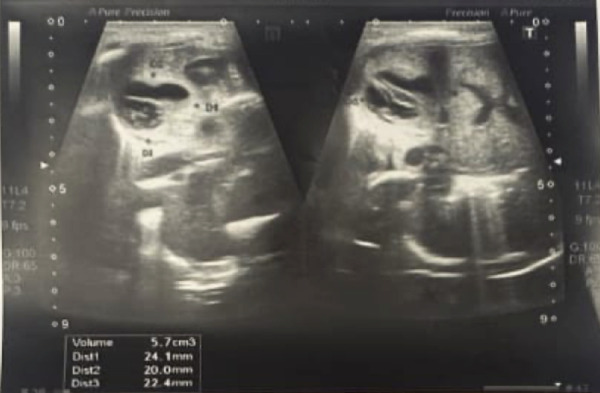
Ultrasound abdomen showing right-sided adrenal haemorrhage

The baby was shifted to the NICU, and an abdominal ultrasound was performed to rule out adrenal haemorrhage. Following the surgical intervention, an abdominal ultrasound was performed to rule out adrenal haemorrhage, considering the presence of bilateral scrotal hematomas. The ultrasound revealed both kidneys to be normal, but a well-defined, lobulated, heterogeneously hypoechoic area with internal echogenic and cystic components was noted at the superior pole of the right kidney, measuring 2.4 × 2.0 × 2.2 cm (5.7 mL). This confirmed the presence of a right-sided adrenal haemorrhage (Figure 5).

Laboratory investigations showed a decline in haemoglobin levels from 16.3 g/dL to 14.1 g/dL, accompanied by a rise in bilirubin levels from 48 µmol/L to 151 µmol/L. The rest of the investigations were within normal limits. The child was managed conservatively with intravenous fluids, antibiotics, oxygen support, coagulation management, nutritional support, and thermal regulation. Adherence to treatment was assessed through serial monitoring, including follow-up ultrasounds, which showed progressive resolution of the adrenal haemorrhage. Gradual symptomatic improvement was observed, and the baby tolerated the interventions well without any postoperative complications. The child was discharged on the 17th postnatal day. The baby did not experience any adverse or unanticipated events postoperatively or during conservative management of adrenal haemorrhage.

The initial diagnosis of neonatal testicular torsion was reconsidered intraoperatively, as the testes appeared normal with bilateral hematomas. The final diagnosis was bilateral scrotal hematomas with an incidental right adrenal haemorrhage. The adrenal haemorrhage was most likely due to birth-related trauma associated with emergency C-section and neonatal resuscitation. The child was managed conservatively and showed complete resolution of both conditions on serial ultrasounds. Follow-up at 1 and 3 months confirmed a thriving infant with no long-term complications. In this case, there were no significant financial or cultural barriers to treatment, as the parents provided informed consent for surgical exploration without hesitation. The parents of the patient were satisfied with the treatments their child received at the time of discharge.

## DISCUSSION

Neonatal adrenal haemorrhage (NAH) is a rare but clinically significant condition, often resulting from perinatal stress or birth trauma. The neonatal adrenal glands are particularly susceptible due to their large size—up to 20 times larger than in adults relative to body weight—and their enhanced vascularity, receiving arterial supply from 50–60 sub-arteries originating from three suprarenal arteries. These anatomical factors, combined with fluctuations in venous pressure during delivery, increase the risk of haemorrhage ^6^.

**Figure 6:**
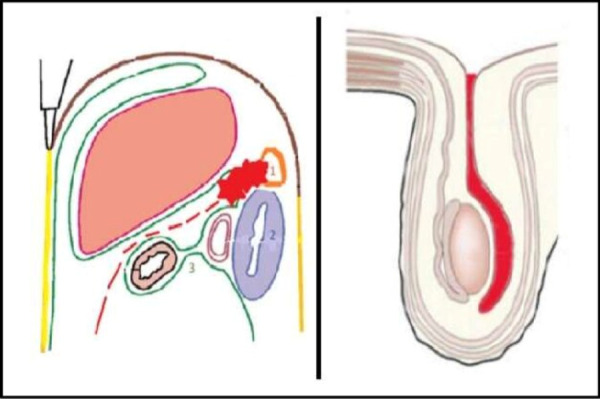
Schematic representation of retroperitoneal adrenal haemorrhage with blood tracking into the scrotum

The clinical presentation of NAH is highly variable, ranging from asymptomatic cases to severe adrenal insufficiency ^7^. A particularly rare presentation is scrotal hematoma or ecchymosis, known as Stabler’s or Bryant’s sign, which occurs when blood from the adrenal gland tracks along the retroperitoneum and inguinal canal (Figure 6)^8^. This phenomenon is attributed to the shared embryological origin of the adrenal glands and gonads from the urogenital ridge. The most significant diagnostic challenge in such cases is differentiating NAH-induced scrotal hematoma from neonatal testicular torsion, as both conditions present with scrotal swelling and bluish discoloration ^9^.

On the left, the diagram illustrates retroperitoneal adrenal haemorrhage (label 1) adjacent to the kidney (label 2), with blood dissecting inferiorly into the peritoneal cavity (label 3). On the right, the illustration shows blood extending downward toward the scrotum, explaining the clinical finding of scrotal hematoma.^10^

Doppler ultrasonography is the first-line diagnostic tool to distinguish between these conditions ^11^. However, in our case, extensive scrotal hematoma obscured Doppler findings, leading to an initial misdiagnosis of testicular torsion and surgical exploration. The intraoperative finding of bilateral scrotal hematoma prompted retrospective abdominal ultrasonography, which confirmed NAH.

A review of the literature indicates that the right adrenal gland is more commonly affected, with birth asphyxia being the most frequently reported underlying insult, consistent with our case. Neonatal jaundice is also a commonly associated comorbidity ^12^. While previous case reports have described scrotal hematomas ipsilateral to the affected adrenal gland or, rarely, on the contralateral side ^13^, our case is unique in that unilateral right-sided NAH resulted in *bilateral* scrotal hematomas.

A systematic review of NAH cases with scrotal hematoma found that only *42 cases* were reported up to 2022, of which *11 (26.2%)* required surgical exploration due to diagnostic uncertainty ^13,14^ (Table 1).

**Table 1:** Summary of Reported Cases of Neonatal Adrenal Haemorrhage Associated with Scrotal Hematoma (1989–2022)

Parameter	Cases (1989–2011)^13^	Cases (2012–2022)^12^	Total Cases
Total Cases	29	13	42
Treatment			
Conservative	20	11	31
Surgical	9	2	11
Side of AH (Adrenal Haemorrhage)			
Right	-	10	10
Left	-	1	1
Bilateral	-	1	1
Unknown	-	1	1
Side of SH (Scrotal Hematoma)			
Right	-	10	10
Left	-	2	2
Bilateral	-	1	1

Other non-scrotal causes of neonatal scrotal hematoma have also been reported, including splenic rupture and subcapsular liver hematoma, both of which can present as life-threatening conditions^15,16^. These findings underscore the importance of considering intra-abdominal pathologies in cases of neonatal scrotal hematoma and maintaining a broad differential diagnosis.

The primary treatment of NAH with scrotal hematoma remains conservative, focusing on supportive care and serial ultrasonographic follow-up ^17,18^. The resolution time depends on hematoma size and volume, as well as overall neonatal health, presence of complications like sepsis, and coagulation abnormalities. Haemorrhages smaller than *10 mL*, with diameters under *2–3 cm*, such as in our case, typically resolve within *two weeks* ^19^.

This case highlights the strengths of early detection and intervention in neonatal scrotal emergencies. Timely imaging and surgical exploration allowed for appropriate hematoma evacuation and prevented unnecessary orchiectomy. Additionally, recognizing the need for abdominal ultrasound led to early identification of adrenal haemorrhage, ensuring conservative management. However, there were some limitations. The initial presentation suggested neonatal testicular torsion, but intraoperative findings revealed bilateral scrotal hematomas instead. While Doppler ultrasound raised suspicion, the final diagnosis was only confirmed surgically. Additionally, adrenal haemorrhage was an incidental finding, which could have been overlooked without further imaging.

This case also highlights the potential limitations of ultrasonography in diagnosing scrotal hematomas, particularly in settings with limited operator experience. However, neonatal testicular torsion can sometimes be a diagnostic challenge, particularly in settings where Doppler ultrasound is not immediately available, especially in a country with limited resources.

This case reinforces the importance of a high index of suspicion for NAH in neonates presenting with acute scrotum, particularly in the presence of risk factors such as birth asphyxia. Early incorporation of abdominal ultrasound in the diagnostic workup can prevent unnecessary surgical interventions and anaesthesia exposure, improving patient outcomes.

## CONCLUSION

Neonatal adrenal haemorrhage should be considered in the differential diagnosis of acute scrotum in neonates, and combined abdominal and scrotal ultrasound can help avoid unnecessary surgery through accurate diagnosis.
